# From flower buds to bolls: how cotton reproductive structures shape boll weevil development, reproduction and survival

**DOI:** 10.1002/ps.70574

**Published:** 2026-01-19

**Authors:** Roberta Ramos Coelho, Guilherme Gomes Rolim, Franklin Magliano da Cunha, Valéria Wanderley‐Teixeira, José Dijair Antonino, Jorge Braz Torres

**Affiliations:** ^1^ Departamento de Agronomia‐Entomologia Universidade Federal Rural de Pernambuco Recife Brazil; ^2^ Instituto Mato‐Grossense do Algodão Cuiabá Brazil; ^3^ Centro Universitário Frassinetti do Recife – UniFAFIRE

**Keywords:** *Anthonomus grandis grandis*, life history, nutrition, vitellogenin

## Abstract

**BACKGROUND:**

The cotton boll weevil *Anthonomus grandis grandis* is a major pest in tropical and subtropical cotton crops, where its management relies heavily on insecticide applications. Despite that, the physiological mechanisms enabling survival and reproduction in the tropical off‐season remain poorly understood. This study examined host usage, and physiological pathways linking nutrition and reproduction in boll weevils, and how larval and adult food sources affect development, survival and reproduction.

**RESULTS:**

Larvae fed bolls extended developmental time, produced larger adults, accumulated greater lipid reserves and survived longer, particularly if fed pollen when adult, but females exhibited significantly reduced fecundity compared with those developing in flower buds. By contrast, flower bud‐fed adults displayed enhanced ovariole development, higher protein content and markedly increased daily egg production. Molecular analysis revealed that *vitellogenin* (*Vg*) expression was reduced in females fed on bolls or cotyledons, suggesting nutritional constraints impairing reproductive capacity. *Vg* expression was 2.5‐fold higher in bud‐fed females relative to boll‐fed counterparts, whereas *FOXO* expression remained unaffected. These results demonstrate a reproduction‐longevity trade‐off driven by adult diet quality: flower buds promoted reproductive output, whereas bolls favored lipid storage and extended survival.

**CONCLUSION:**

Feeding on cotton flower buds sustains continuous reproduction in *A. grandis grandis*, whereas boll‐based diets reduce vitellogenesis and favor survival through lipid accumulation. This nutritional shift explains how tropical populations persist during host‐free periods but resume reproduction once they return to flower bud feeding. The findings highlight the need for strict crop‐free intervals and provide a physiological basis for refining integrated management strategies. © 2026 The Author(s). *Pest Management Science* published by John Wiley & Sons Ltd on behalf of Society of Chemical Industry.

## Graphical abstract

Female boll weevils feeding on flower buds promote reproduction regardless of their immature diet, whereas boll feeding increases lipid reserves and lifespan, revealing a nutrition‐driven trade‐off that sustains survival and reproduction in tropical boll weevil populations.

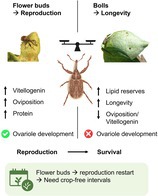

## 1 INTRODUCTION

The cotton boll weevil, *Anthonomus grandis grandis* Boheman (Coleoptera: Curculionidae), is the most damaging cotton pest in subtropical and tropical regions.^1^, ^2^ In Brazil, where ≈2 million ha of cotton is cultivated each season, an average 19.6 insecticide applications have been used to manage boll weevils even though several proactive management strategies have been adopted against this pest.^3^ Despite integrated efforts, management strategies remain largely dependent on broad‐spectrum insecticides. This approach leads to an estimated cost of US$360 ha^−1^ (for boll weevil management alone), excluding indirect costs. This involves ≈20 insecticide applications per season, which negatively affects natural enemy populations, creating a feedback loop between reliance on insecticide spraying and the prevention of natural enemies' establishment.^4^, ^5^

Boll weevil damage results from feeding, oviposition and larval development in cotton reproductive structures, leading to fruit abscission and fiber loss.^6^, ^7^ During the off‐season, after crop residues are removed, adults persist in low reproductive states by feeding on alternative hosts or survive imprisoned in dry bolls,^7^, ^8^, ^9^ despite legislation establishing a time period with no viable host plant material in the field, also called the sanitary break.^10^

In temperate regions, cold winter temperatures combined with the cessation of cotton flowering, cause dispersal to overwintering sites, resulting in substantial off‐season mortality and a trade‐off between reproduction and survival, enabling eradication campaigns.^11^ By contrast, tropical environments allow part of the population to remain active during the off‐season.^8^, ^12^, ^13^ During the harvesting period, adults disperse to other areas where they survive by feeding on other plants' fruiting structures, but they do not reproduce.^14^, ^15^, ^16^ Reproduction in these areas appears strongly linked to cotton phenology, with suppressed reproductive activity when adults feed mainly on small bolls.^17^ This pattern may reflect nutritional constraints. Furthermore, in the Brazilian Midwest, boll weevil reproduction appears to be tightly related to cotton phenology: when small bolls prevail, a higher percentage of males and females deviate from their physiological reproductive conditions to survive the off‐season.^17^ The absence of reproduction could be attributed to an inferior nutritional intake, as flower buds contain more protein than bolls, and protein, amino acids and lipids are important precursors of hormones that promote reproductive system development.^18^, ^19^

Cotton flower buds and bolls differ fundamentally in structure and composition. Flower buds are short‐lived tissues supporting rapid growth with higher protein content, yet bolls are the plant's final fruiting stage, containing protein‐ and oil‐rich seeds encased by a fibrous carbohydrate‐rich wall.^20^ Comparative studies in other plant species show that flower buds can contain higher sterol and phospholipid levels than fruits,^21^ nutrients that serve as hormone precursors.^22^, ^23^, ^24^ Such biochemical differences suggest that larval and adult diets based on buds or bolls may have contrasting effects on insect physiology and reproduction. Nutrition is a key regulator of insect reproduction, primarily through its influence on vitellogenesis, which depends on vitellogenin (Vg) synthesis. Vg is the major yolk‐protein precursor in developing oocytes, and vitellogenesis is mainly regulated by juvenile hormone (JH) and the ecdysteroid 20‐hydroxyecdysone (20E).^25^, ^26^, ^27^ Hormone synthesis is further modulated by nutritional sensors such as amino acid/Target of Rapamycin (AA/TOR) and insulin/ insulin‐like peptide (ILP) pathways.^22^, ^26^, ^27^, ^28^, ^29^ Forkhead box O (FOXO) proteins also integrate the response to starvation, functioning as transcription factors that regulate stress resistance,^30^ *vg* gene expression and diapause.^31^, ^32^

Despite all of this, the physiological mechanisms underlying boll weevil survival and reproduction during the off‐season in tropical regions remain poorly understood. Here, we tested the hypothesis that feeding on bolls can trigger physiological traits related to reproduction in boll weevils from tropical areas. Specifically, we evaluated the effects of flower buds *versus* boll diets during larval and adult stages on biology and reproductive traits, with particular emphasis on how larval and adult diets influence female reproductive function.

## 2 MATERIAL AND METHODS

### 2.1 Insects

Cotton flower buds bearing oviposition punctures were collected from experimental microplots at the Universidade Federal Rural de Pernambuco (UFRPE), Recife, Brazil, and maintained in plastic trays inside acrylic cages (30 × 45 × 50 cm) at the Laboratory of Biological Control (UFRPE) until adult emergence. Adults were sexed,^33^ placed individually in 80‐mL plastic pots, and fed daily with cotton cotyledons and flower buds for 4 days to ensure sexual maturation.^34^ The 4‐day‐old adults were randomly paired for 2 days to allow mating. Females were then transferred to caged cotton plants bearing either flower buds or bolls, with the alternative structures removed. These females produced the F_1_ generation used in the subsequent assays.

### 2.2 Larval food source effects on development and reproduction

Cotton plants (~60 days old) were prepared by removing either all bolls or all flower buds, depending on the treatment. Plants were enclosed in cylindrical mesh cages (1.0 m diameter × 1.20 m height) with Velcro™ side openings. Three mated females (6 days old) were allowed feeding and laying eggs freely on the accessible reproductive structures. Following this period, the females were removed, and the plants were checked daily for falling buds and oviposited bolls. Because attacked bolls do not usually abscise,^35^ thus those that showed evidence of oviposition were collected on Day 15 day after the females were confined.

The collected structures—fallen buds and bolls removed from the plants—were taken to the laboratory for individual monitoring of F_2_ adult emergence. These adults were categorized into males and females, and the time for immature development was recorded. This variable represents the duration between the release of F_1_ females in the cages and the emergence of F_2_ adults from the affected structures. Additionally, the fresh body mass (in mg) of the emerged male and female adults was measured using a precision balance with an accuracy of 0.0001 g.

In order to assess ovipositiion performance, F_2_ females emerged from flower buds or bolls were released into cages enclosing cotton plants bearing only flower buds or developing bolls. This setup was established using a completely randomized experiment with a two‐way arrangement consisting of two main factors represented by F_2_ female origin (flower buds or bolls) and two levels (oviposition on flower buds or bolls), totaling three treatments: (i) F_2_ females emerged from flower buds released on plants with flower buds; (ii) F_2_ females emerged from flower buds released on plants with bolls; and (iii) F_2_ females emerged from bolls released on plants with flower buds. Each treatment was represented by six microplots, each of which included three cotton plants. Three days after the female release, the insects were collected and the plants checked for oviposition.

### 2.3 The larval food source and boll weevil longevity

In order to evaluate the influence of larval food source on the adult longevity, adult males (F_2_ generation) originating from either flower buds or bolls were released into cages containing three plants with either buds (~6 mm) or bolls (10–15 mm diameter). Females remained confined for 3 days to feed and to lay eggs. After removal, plants were monitored daily for abscised buds and oviposited bolls;^35^ bolls were collected 15 days later.

Collected structures were taken to the laboratory, and F_2_ adults were sexed. Developmental time (from F_1_ female release to F_2_ adult emergence) and adult body mass (mg, precision 0.0001 g) were recorded. To assess oviposition, F_2_ females emerged from buds or bolls were released into cages with plants bearing either buds or bolls, resulting in four treatments in a two‐way factorial design. Each treatment was replicated six times (microplots with three plants each). Females were removed after 3 days, and oviposition was recorded.

Male and female F_2_ adults emerged from either flower buds or bolls were marked dorsally with water‐based paint for identification. Pairs were maintained in Petri dishes (15 × 90 mm) under three adult diets: (i) cotton pollen + water, (ii) cotyledons + water, or (iii) water only. Water was provided on moistened cotton, and pollen (Breyer®; União da Vitória, PR, Brazil) was supplied in microtube caps. Each treatment included 10 males and 10 females. The study followed a 2 × 3 factorial design: two larval food sources (flower buds or bolls) and three adult diets, totaling six treatment combinations. Insects were maintained at 26 °C under a 12 h:12 h, light:dark photoperiod, with food replaced every 2 days. Mortality was recorded daily and represented as survival curves.

### 2.4 Larval and adult food effects on ovariole development

Newly emerged females from field‐collected bolls were fed for 5 days on flower buds, bolls or cotyledons (15 females per treatment). Females were then chilled (4 °C) and dissected under a stereomicroscope to examine ovariole morphology. Ovarioles were fixed in 10% formalin, gradually dehydrated in ethanol, embedded in Leica® historesin for 24 h, sectioned (3 μm), and stained with haematoxylin & eosin. Five ovarioles per treatment were analyzed, each considered as a replicate. Protein content was determined using Xylidine,^36^ and glycogen detection was performed with the PAS (periodic acid Schiff) technique.^37^ Slides were photographed, and the images were analyzed using GIMP 2.0 software by measuring pixel intensity.

### 2.5 Biochemical analysis of F_2_ females

The F_2_ females (*n* = 10 per treatment) emerged from either flower buds or bolls and fed the same structures for 5 days were used for biochemical assays. Soluble protein, lipids, sugar and glycogen were quantified in the Histology Laboratory of UFRPE.

For soluble proteins, samples were prepared in carbonate buffer [15 mm sodium carbonate (Na₂CO₃), 35 mm sodium bicarbonate (NaHCO₃), pH 9.6) and centrifuged at 4 °C and 10 000 rpm for 15 min. Protein quantification followed the Bradford method (1976), mixing the supernatant with 5 mL Bradford reagent (0.01% Coomassie Brilliant Blue G‐250, 8.5% phosphoric acid, 4.7% ethanol). Absorbance was read at 595 nm in a spectrophotometer.

Lipids, sugars and glycogen were extracted by chloroform: methanol partitioning. Organic (lipids) and aqueous (sugars) phases were quantified using sulfuric acid‐vanillin (lipids) or anthrone reagent (sugars and glycogen), with absorbance read at 625 nm.^38^, ^39^

### 2.6 Gene expression of *vitellogenin* (*Vg*) and *forkhead box O* (*FOXO*)

Females emerged from field‐collected flower buds were fed for 5 days on either buds or bolls. Each treatment consisted of three biological replicates of four females each, and the experiment was repeated twice. Total RNA was extracted using TRIzol® reagent (Invitrogen/Thermo Fisher Scientific, Waltham, MA, USA) according to the manufacturer's protocol. Genomic DNA was removed with 2 U RNase‐free DNase I (Ambion^/Thermo Fisher Scientific^). RNA integrity was confirmed by 1% agarose gel electrophoresis, and concentrations were determined with a spectrophotometer. cDNA synthesis was performed with 1 μg total RNA using GoScript™ reverse‐transcriptase (Promega, Madison, WI, USA), following the manufacturer's instructions.

Quantitative reverse transcription (qRT)‐PCR) was conducted on a performed using a QuantStudio 5 machine (Thermo Fisher Scientific) using SYBR Green GoTaq® qPCR Master Mix and primers specific for *Vg* and *FOXO* (Table 1). Cycling conditions were: incubation at 95 °C for 10 min, followed by 40 cycles of 95 °C for 15 s and 60 °C for 1 min, with melting curve analysis. *GAPDH* (GABY01019565) and *beta‐tubulin* (GABY01011644) were used as reference genes^34^ (Table 1).

**Table 1:** Primer sequences (5′ – 3′) and target genes used in this study

Name	Sequence 5′ – 3′	Target gene
AgraGAPDHFor	AGATCGTCGAGGGTCTGATG	*GAPDH*
AgraGAPDHRev	AAGGCGGGAATGACTTTACC	
AgraBtubFor	GGTTGCGACTGTTTACAAGG	*β‐tubulin*
AgraBtubRev	GCACCACCGAGTAAGTGTTC	
AgraVgFor	TCATCAAATCTATATGGCTGGTTATGAC	*Vitellogenin*
AgraVgRev	GCTACAGGACTAATTGCCATAACATCAC	
AgraFOXOFor	GACGACCCAGGCAAAGGTAA	*Forkhead box O*
AgraFOXORev	TGGTAGGGCGGGATTGTAGA	

### 2.7 Statistical analysis

All analyses were conducted using the SAS software package.^40^ The duration of the immature stage, the adult fresh body mass of F_2_ males and females, and oviposition rates (see Section 2.2) were analyzed using a generalized linear model (GLM) procedure after assessing the data for normality and homogeneity of variance. To satisfy the assumptions of ANOVA, the data were log‐transformed using either log(*x*) or log(*x* + 0.5). Following ANOVA, if significant differences were found, treatment means were compared using Tukey's honestly significant difference (HSD) test at a 5% significance level. Additionally, daily mortality rates recorded for females and males (see Section 2.3) were pooled to construct survival curves using the Kaplan–Meier method, which were then compared using the log‐rank test (PROC LIFETEST). Data on total protein, lipid, sugar and glycogen obtained using the method in Section 2.5 also were tested for normality and homogeneity of variance. For lipid data collected from field insects, square‐root transformation (*x* + 0.5) was necessary. The means among treatments were compared using Tukey's HSD test at the 5% significance level. *Vg* transcript expression was quantified based on the number of cycles needed to reach a predetermined threshold during the exponential phase of PCR (Cq values), following RDML standards.^41^ Relative expression was calculated using the ΔΔCt method,^42^ with qbase+ software (Biogazelle, Ghent, Belgium).

## 3 RESULTS

### 3.1 Larval food source effects on development and reproduction

Developmental time, adult fresh body mass and oviposition were evaluated in relation to larval food source, sex and F_1_ origin, including their interactions. Only larval food source significantly affected developmental time (*F*₁, ₂₃₀ = 1100.09, *P* < 0.0001), adult fresh body mass (*F*₁, ₂₀₃ = 96.42, *P* < 0.0001) and oviposition rate (*F*₁, ₁₅ = 200.92, *P* < 0.0001). Weevils developing in bolls required ~15 additional days to reach adulthood compared with those developing in flower buds (Table 2). Adults emerging from bolls were 1.7‐fold larger than those from flower buds (Table 2). By contrast, F_2_ females laid nearly twice as many eggs per day when confined to plants bearing only flower buds compared with plants bearing only bolls (Table 2). These results demonstrate that the food source strongly influenced development, body size and reproduction.

**Table 2:** Developmental time of *A. grandis grandis* larvae, adult fresh body weight and number of eggs per female per day according to the food source of offspring, related to F_1_

F_1_ ^†^	F_2_ ^‡^	Developmental duration (days)	Adult weight (mg)	Number of eggs/ female/ day
Flower buds	Flower buds	14.8 ± 0.31b	7.8 ± 0.36b	2.6 ± 0.08a
	Bolls	29.1 ± 0.64a	13.1 ± 0.59a	1.3 ± 0.07b
Bolls	Flower buds	14.7 ± 0.21b	7.5 ± 0.37b	2.4 ± 0.11a
	Bolls	28.1 ± 0.54a	12.9 ± 0.81a	1.3 ± 0.04b

^†^ There was no effect of parental origin on the evaluated characteristics (*P* > 0.05).

^‡^ Means (+SE) followed by the same letter, in the column, do not differ by the Tukey's honestly significant difference (HSD) test at 5% probability.

### 3.2 Larval food source and adult boll weevil longevity

**Figure 1:**
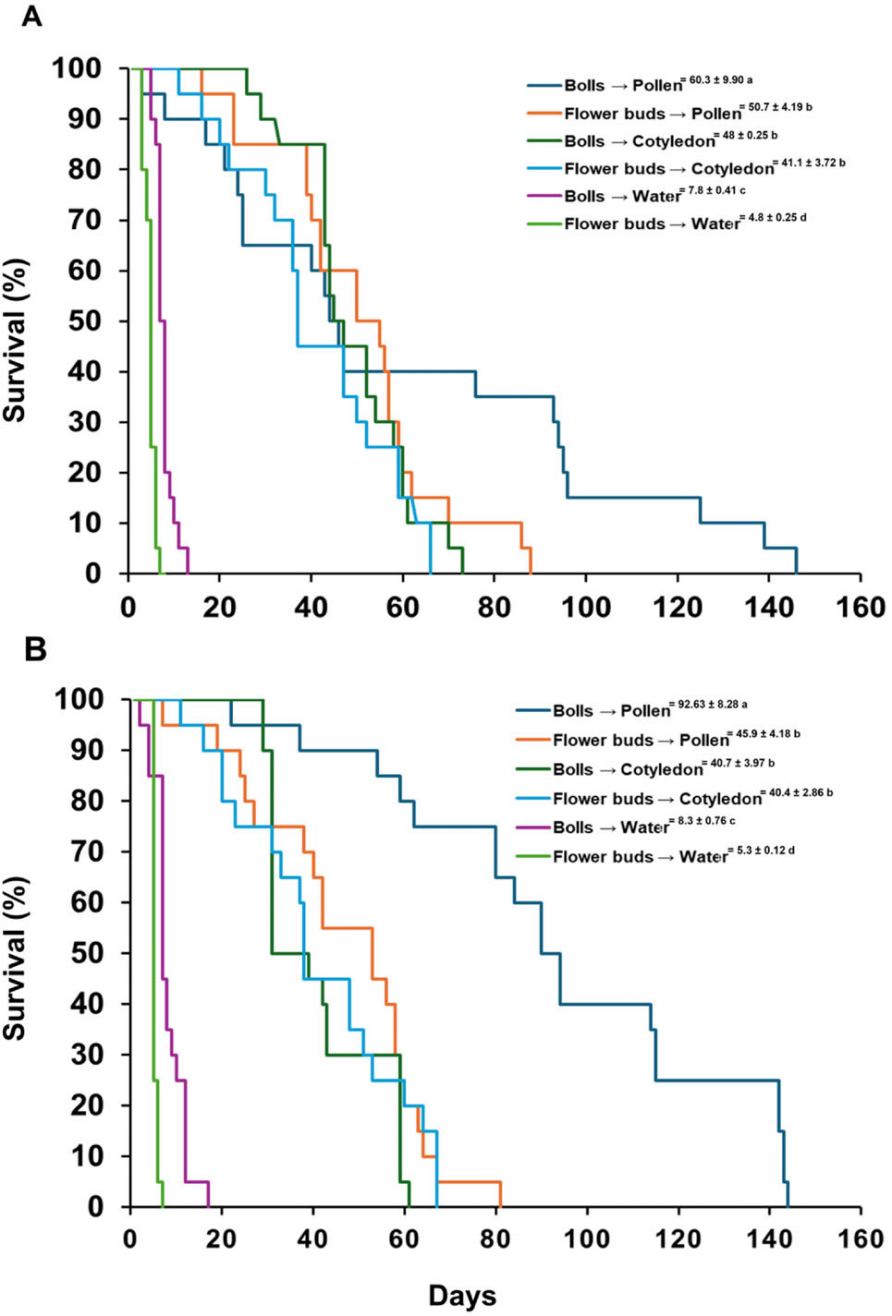
Survival of *A. grandis grandis* adults emerged from the greenhouse generation (F_2_) (A) and from attacked field‐collected bolls or flower buds at the end of the growing season (B) and fed with pollen + water, cotton cotyledons + water, or only water. The means ± SE of survival were estimated using the Kaplan–Meier method and compared using the log‐rank test at a 5% probability level.

The influence of food sources utilized by immature on insect survival is well‐established.^43^ To explore the topic further, we simulated how larval food and adult diet affects adult boll weevil survival. Survival did not differ between males and females (*P* > 0.05), so data were pooled. However, both the origin of the adults (from flower buds or bolls) and the alternative food provided during adulthood significantly affected survival in greenhouse‐reared [log‐rank; *χ* ^2^ = 8.06, *P* = 0.0447, df = 5; Fig. 1(A)] and field‐collected adults [*χ* ^2^ = 199.35, *P* < 0.0001, df = 5; Fig. 1(B)].

Adults emerging from bolls and fed pollen survived the longest; with some individuals living >120 days under both greenhouse and field conditions (Fig. 1). Even when fed only water, adults from bolls lived longer than those from flower buds. Pollen consistently extended survival more effectively than cotton cotyledons (Fig. 1).

### 3.3 Food source effects on ovariole development

**Figure 2:**
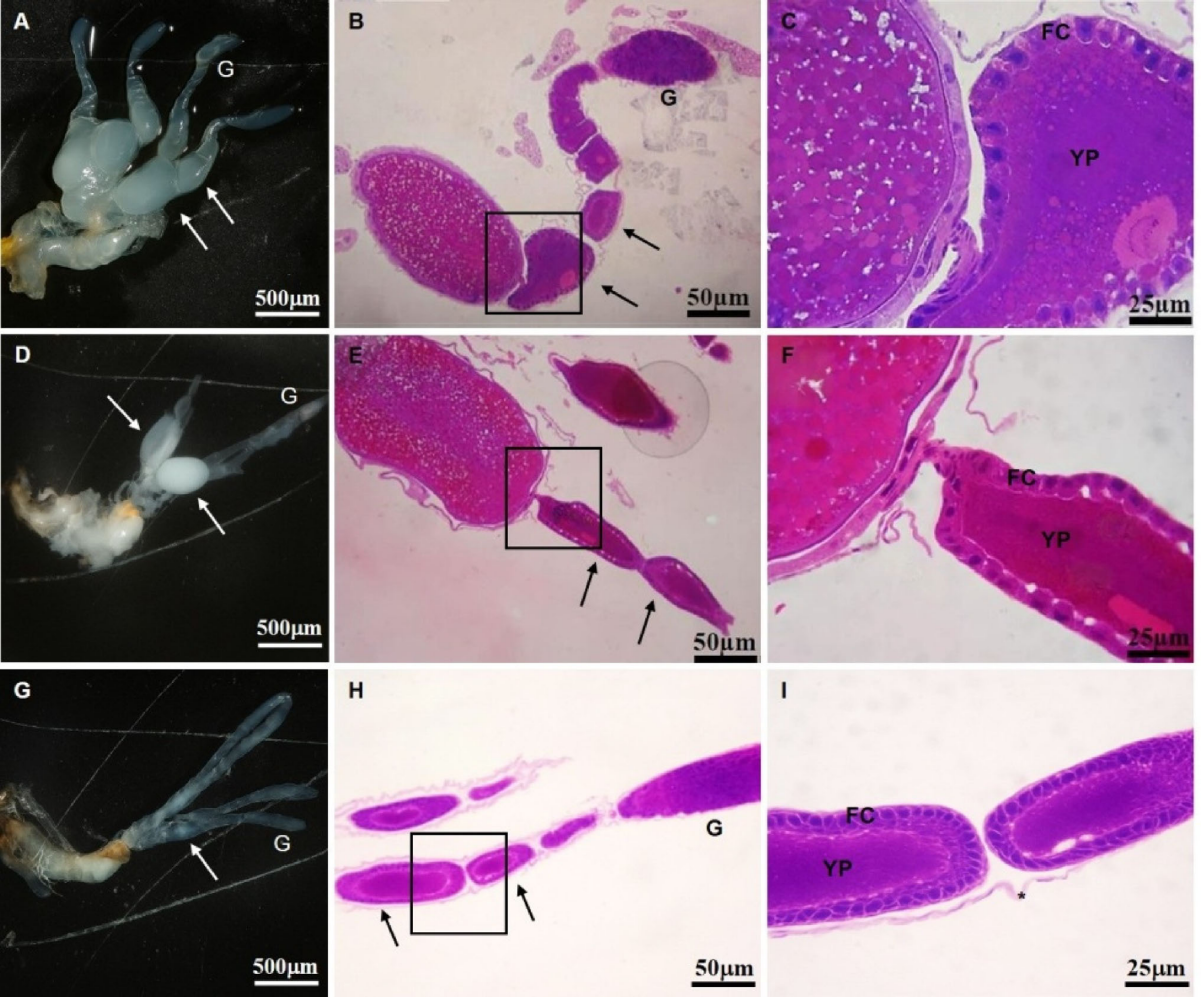
Ovarioles extracted and histological sections from females of *A. grandis grandis* emerged from bolls and fed with flower buds (A–C), fed bolls (D–F) and fed cotyledons (G–I) for five consecutive days after emergence. In (A–C) the ovarioles show a well‐developed germarium (G) and vitellarium area with the presence of oocytes in various stages of maturation (arrows). In (D–F), the vitellarium has a smaller number of developing oocytes, and (G–I) ovarioles of females fed cotyledons, showing poorly developed vitellarium. It is noted in all treatments that no histological changes occurred in the ovarioles, which presented a thin layer of connective tissue (asterisks) surrounding the well‐preserved germarium (G) and vitellarium; developing oocytes (black arrows) can be observed. Follicular cell (FC) and yolk protein (YP). Hematoxylin & eosin.

Females emerging from mature bolls and fed different food sources for 5 days exhibited clear differences in ovariole development. Females fed on cotyledons displayed smaller ovariole and fewer developing oocytes than those fed on flower buds or bolls (Fig. 2). Females fed on bolls [Fig. 2(D)–(F)] also showed impaired ovariole development compared with those fed on flower buds [Fig. 2(A)–(C)]. By contrast, females fed on flower buds developed well‐structured ovarioles with oocytes at multiple stages [Fig. 2(A),(B)]. Histological sections confirmed yolk protein deposition and developing oocytes in females fed on flower buds, whereas yolk was absent or markedly reduced in females fed on bolls [Fig. 2(E),(F)] or cotyledons [Fig. 2(H),(I)].

### 3.4 Biochemical analysis of F_2_ females

**Figure 3:**
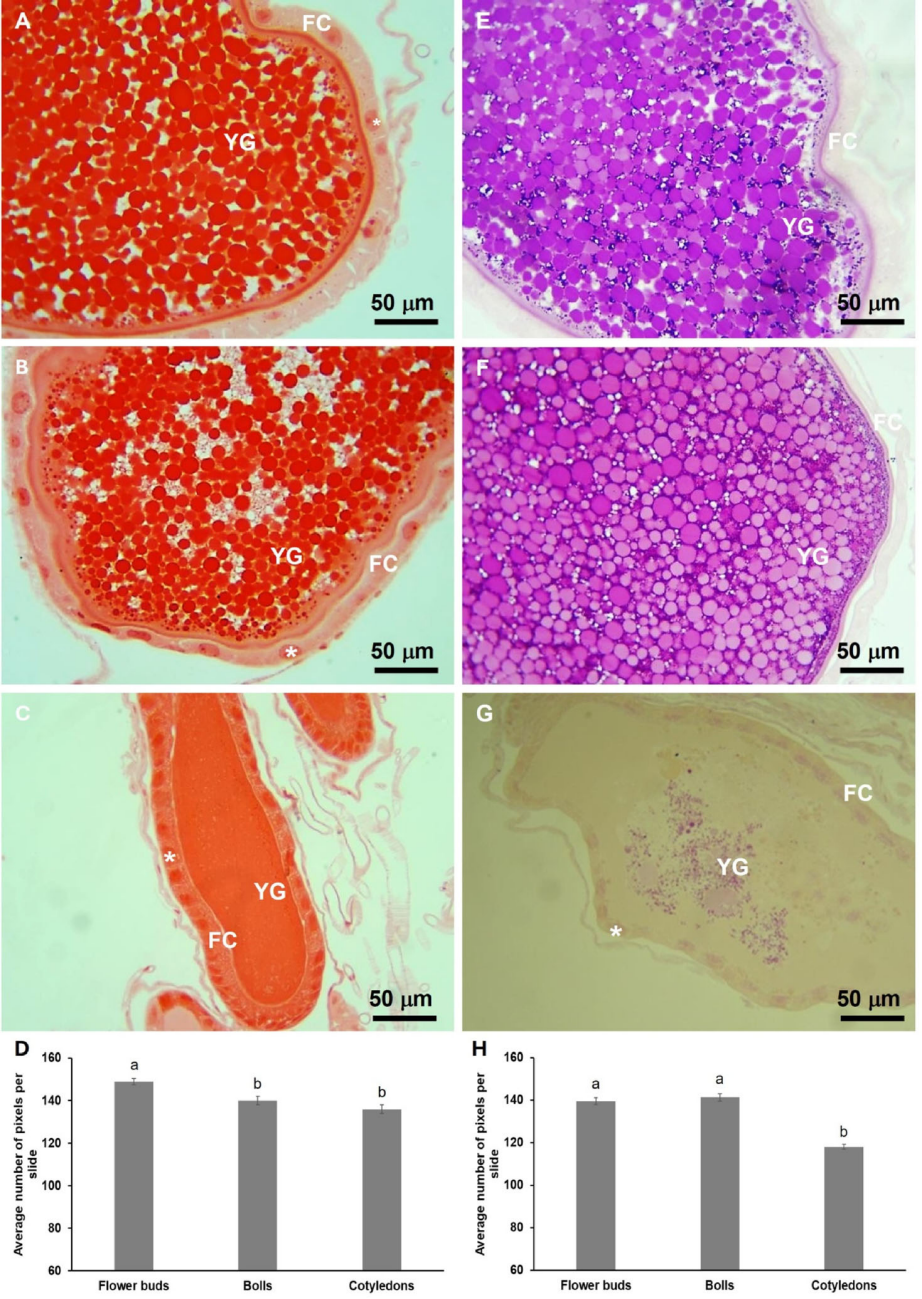
Histochemistry of the *A. grandis grandis* ovarioles obtained from females emerged from bolls and reared for five consecutive days feeding on flower buds (A and E), bolls (B and F) or cotyledons (C and G). Total proteins were determined using Xylidine Ponceau (A‐C) and the glycogen content was determined using the PAS technique staining neutral polysaccharides (E–G). The average pixel count for total proteins (D) and neutral polysaccharides (H) were obtained from histochemical analyses. The observed structures include yolk granules (YG), follicle cells (FC) and nuclei (*).

Histochemical analyses of ovarioles revealed significantly higher protein content in females fed flower buds compared with other food sources (*F*₂, ₆ = 9.58, *P* = 0.0021) [Fig. 3(A)–(D)]. Biochemical assays confirmed these results, showing higher total protein levels in females fed on flower buds then in those fed on bolls (*F*₃, ₃₆ = 9.35, *P* < 0.0001). No significant differences in lipid content were observed among greenhouse‐reared adults (*F*₃, ₂₄ = 0.85, *P* = 0.4810). However, field‐collected females emerging from mature bolls at the end of the cotton season contained 2.1‐fold more lipids (502.4 ± 79.78 μg mL^−1^) than females from flower buds (238.9 ± 22.91 μg mL^−1^) (*F*₃, ₂₉ = 7.6, *P* = 0.0007) (Table 2). No significant differences were observed among males (*P* > 0.05).

Histochemical analysis showed that females fed on flower buds or bolls accumulated more glycogen than those fed on cotyledons (*F*₂, ₆ = 34.7, *P* = 0.0025) [Fig. 3(F),(G)]. However, quantitative assays revealed no significant differences in total sugars or glycogen between adults derived from buds or bolls, under either greenhouse or field conditions (*P* > 0.05). Sugar levels ranged from 23.9 to 37.2 μg mL^−1^ and 38.5 to 55.5 μg mL^−1^ in greenhouse‐reared insects and field‐collected individuals, whereas glycogen levels ranged from 71.8 to 101.4 μg mL^−1^ and 64.92 to 92.5 μg mL^−1^, respectively.

### 3.5 Food source effects on *Vg* and *FOXO* expression

**Figure 4:**
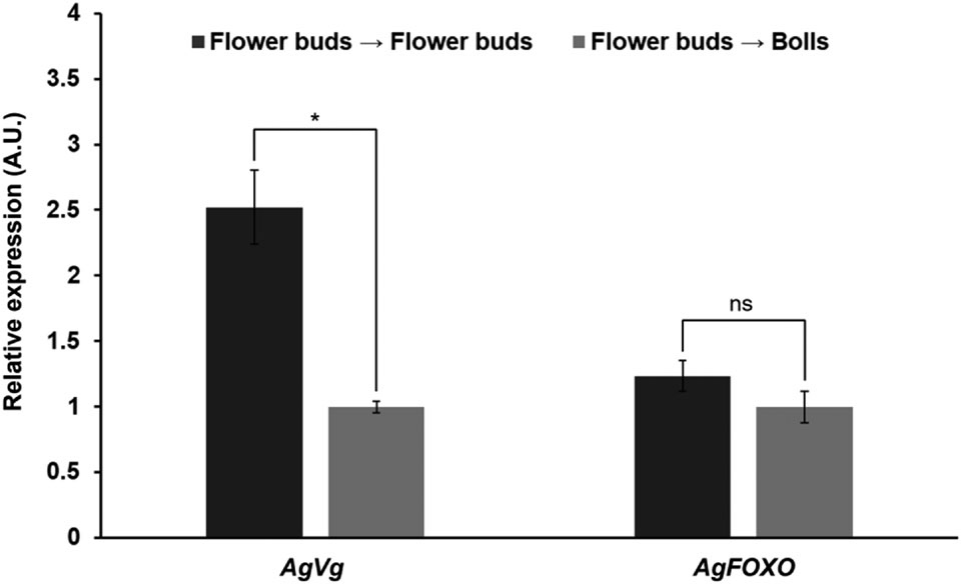
Relative expression of *vitellogenin* and *FOXO* in *A. grandis grandis* females from flower buds and reared for 5 days on flower buds (flower buds > flower buds) or bolls (flower buds > bolls). A.U., arbitrary unity. Significance: *, *P* < 0.05.

*Vitellogenin* expression also was measured in females fed on flower buds (baseline) and bolls. *Vg* expression was significantly influenced by adult diet. Females fed on bolls for 5 days exhibited a 2.5‐fold reduction in *Vg* compared with those fed on flower buds (Fig. 4). By contrast, *FOXO* expression did not vary with adult diet. Additionally, The C_q_ values for the reference genes were highly stable across all dietary treatments (mean C_q_ ± SD): *GAPDH* (19.67 ± 0.37, CV = 1.88%) and *beta‐tubulin* (21.50 ± 0.76, CV = 3.53%). These results confirm the suitability of these genes as internal controls for our expression analysis.

## 4 DISCUSSION

The results reveal that, although the larval food source influences the development and survival of *A*. *grandis grandis*, adult access to a nutritionally adequate diet is a key determinant of reproductive success. Weevils developing in cotton bolls exhibited prolonged development and increased body mass compared with those developing in flower buds. However, larger body mass did not translate into higher fecundity for boll‐reared female boll weevils. Boll‐reared females laid significantly fewer eggs than flower bud‐reared females. The result indicates that boll nutrition supports weevil development but limits its reproductive output, a trade‐off also observed in other Coleoptera under nutritionally imbalanced diets.^44^, ^45^, ^46^

Adult survival was shaped by both larval and adult diet. Weevils from bolls lived longer than those from buds, even consuming only water, possibly as a consequence of their higher lipid reserves. It is important to mention that cotton seeds contain high levels of lipids, and these lipids also must constitute a food source for boll weevil larvae. Field‐collected boll‐reared females contained twice as much lipid as bud‐reared females, consistent with their enhanced resilience under a different food resource. Lipid accumulation is a central mechanism for sustaining basal metabolism during starvation or diapause,^47^ and thus boll‐reared individuals may be better prepared to withstand the nutritional gaps during off‐season. Pollen, a protein‐ and lipid‐rich resource,^48^ consistently prolonged survival more than cotyledons, confirming that boll weevils can exploit pollen from different plants to persist between cotton seasons, as suggested by previous studies.^8^, ^46^ It is important to note that pollen includes fatty acids and phytosterols, critical precursors for hormone synthesis.^24^

By contrast, traits associated with the development of bud‐reared insects indicated that this source is optimal for reproduction. Bud‐reared females developed functional ovarioles containing multiple oocytes, accumulated higher protein levels and achieved markedly higher daily egg production. This outcome aligns with the known feeding behavior of adults, which preferentially consume buds that are rich in protein and phytosterols,^21^ which are critical nutrients for vitellogenesis.^23^, ^24^ Thus, a flower bud diet appears to provide the precise nutrient necessary to sustain continuous egg production. Remarkably, even boll‐reared females quickly attained reproductive competence after being fed buds for only 5 days, underscoring the strong plasticity of the adult stage. This fact explains why females can continue to reproduce off‐season when they have access to flower buds from cotton regrowth and spontaneous plants alongside other crops.^13^

Therefore, the logical question is how flower buds (squares) and bolls differ in their macronutrient composition, especially proteins and carbohydrates. According to Deans *et al*.,^20^ flower buds have a protein‐to‐carbohydrate (P:C) ratio of ≈1:1.5, whereas bolls have a ratio of ≈1:3 under both greenhouse and field conditions. From this, we can infer that flower buds are proportionally richer in protein (% of dry weight) than bolls. However, one might question this conclusion given that a boll consists of seeds (high protein), lint (almost pure cellulose/carbohydrates) and the rind (high fiber/carbohydrates).^20^ Because the boll weevil larva feeds primarily on the seeds, how can we be sure it has less access to protein? We hypothesize that flower buds provide a more bioavailable, protein‐dense resource than bolls, because the flower bud is a homogeneous resource of high‐protein meristematic tissue, whereas the boll (seed) is a heterogeneous resource in which protein is sequestered behind mechanical barriers (seed coats) and diluted by the accumulation of storage lipids and structural carbohydrates (lint). At the molecular level, reproduction was closely linked to the availability of proteins and sterols. Females reared on flower buds showed 2.5‐fold higher *Vg* expression than those reared on bolls. Additionally, females fed on cotyledons had impaired ovarian development. By contrast, *FOXO* expression remained unchanged across treatments. Our initial hypothesis was that the impairment of vitellogenesis was mediated by a macronutrient deficiency signaled via the ILP pathway resulting in FOXO^49^, ^50^, ^51^ inhibitions of *Vg* expression. However, our results did not support this, at least at the time point examined. Under nutrient‐poor conditions, insulin/insulin‐like peptides (ILPs) are generally downregulated, leading to FOXO dephosphorylation and its movement into the nucleus, where it binds *Vg* promoter elements and suppresses transcription. Conversely, in nutrient‐rich conditions, ILPs are upregulated, causing FOXO phosphorylation and export to the cytoplasm, which reduces repression during vitellogenesis.^51^, ^52^ However, *Vg* expression is not solely controlled by FOXO. The juvenile hormone (JH) pathway also regulates *Vg* transcription in many insects through its nuclear receptor, Methoprene‐tolerant (Met), which forms an activator complex that promotes Vg production.^51^, ^53^ Future transcriptomic analyses at different time points are required to further clarify the details of Vg regulation during the off‐season physiological state of the boll weevil. In this context, previous morphological and histochemical observations^54^ provide an important structural basis for interpreting these molecular results. These authors demonstrated that the fat body of *A. grandis grandis* is composed of metabolically active trophocytes, rich in lipids, glycogen and proteins, showing intense biosynthetic activity consistent with Vg synthesis and secretion. The cellular organization and accumulation of energy reserves described by da Cunha *et al*.^52^ support the idea that nutritional alterations, such as those observed in females fed on bolls, directly affect the functional capacity of this tissue, leading to reduced Vg production and impaired vitellogenesis.

As mentioned before, our findings suggest that, in the boll weevil, FOXO does not directly control vitellogenesis under the tested dietary conditions. Other nutritional factors directly related to reproduction may be involved, such as specific proteins, essential amino acids, or secondary metabolites that are present in flower buds but absent from bolls. An alternative explanation for the regulation of vitellogenesis involves phytosterols. In herbivorous insects, reproductive success appears heavily dependent on the availability of these compounds, as they are the primary precursors for the synthesis of essential hormones, such as ecdysteroids.^23^, ^24^ Consequently, phytosterols may represent a critical nutritional factor influencing the reproductive fitness of *A. grandis grandis*. However, this hypothesis requires further experimental validation. Instead of investing in reproduction, boll‐reared females redirected resources toward lipid storage, as evidenced by their higher lipid content and reduced *Vg* expression. The fat body, the central metabolic organ for both Vg synthesis and lipid storage,^23^, ^45^ appears to shift its metabolic flux from vitellogenesis to lipogenesis when key nutrients are limited. This metabolic reprogramming favors long‐term survival over immediate reproductive output. Downregulation of *Vg* also may represent an adaptive strategy to conserve amino acids and energy for other physiological processes, including immunity and stress responses.^45^, ^53^, ^54^ Indeed, in the mediterranean fruit fly, *Ceratitis capitata* (Diptera: Tephritidae), experimental data and numerical simulations suggest that fecundity increases to the detriment of lifespan when females switch from a sugar‐only diet to one containing protein.^55^ This ability to reallocate resources is an important adaptation that increases insect fitness in a changing environment.

Together, these findings reconcile two paradoxical aspects of boll weevil ecology in tropical regions. On the one hand, boll‐reared adults accumulate lipid reserves and survive for extended periods, enabling persistence despite management practices such as short host‐free periods. On the other, reproduction is markedly reduced under boll diets, constraining population growth until the next flowering season. Importantly, feeding on buds, regardless of larval origin, is sufficient to restore reproductive competence, suggesting that host phenology synchronizes reproduction in field populations.

For a management perspective, the ability of boll‐reared weevils to survive for extended periods on alternative hosts or minimal key resources highlights the need for strict enforcement of cotton‐free intervals >120 days and elimination of cotton fruiting structures after harvest from crop remains, regrowth or spontaneous plants. At the same time, their reduced reproductive potential under boll‐reared diets highlights a vulnerability that could be exploited in integrated management programs by targeting adult survival during the off‐season rather than relying exclusively on chemical control during the growing season.

## 5 CONCLUSION

This study presents the first comprehensive experimental evidence that the adult food source in *A. grandis grandis* serves as a developmental switch. Feeding on flower buds resulted in insects richer in protein, fostering a high‐fecundity reproductive phenotype that is well‐suited to take advantage of peak resource availability. Conversely, development in late‐season bolls leads to low‐fecundity, long‐lived insects marked by reproductive arrest and significant lipid accumulation, which is pre‐adapted for surviving the off‐season. We show that this trade‐off between reproduction and longevity is governed by a shift in resource allocation from protein‐dependent vitellogenesis to lipogenesis, as supported by nutritional, physiological and molecular evidence. These findings provide a new perspective on the seasonal ecology of a major cotton pest and establish a solid foundation for improving integrated management strategies of boll weevil in tropical and subtropical areas.

## FUNDING INFORMATION

The authors declare they have no competing financial interests.

## AUTHOR CONTRIBUTION

**Roberta R. Coelho:** Conceptualization, investigation, visualization, writing, and data curation. **Guilherme G. Rolim:** Investigation, formal analysis, data curation, writing. **Franklin M. Cunha:** Investigation, formal analysis, writing. **Valéria Wanderley‐Teixeira:** Conceptualization, writing – review and editing. **José D. Antonino:** Conceptualization, visualization, writing and review. **Jorge B. Torres:** Conceptualization, investigation, writing, funding acquisition, project administration. All authors read, corrected, and approved the final version.

## CONFLICT OF INTEREST

The authors declare they have no conflicts of interest.

## INFORMED CONSENT

The authors recognize that the paper is being submitted for publication in the *Pest Management Science*, and assure that this paper has not been submitted, accepted, or published in another journal.

## ETHICAL APPROVAL

This article does not contain any studies with human participants performed by any of the authors.

## Data Availability

The data that support the findings of this study are available from the corresponding author upon reasonable request.
